# The prognostic role of urea-to-creatinine ratio in patients with acute heart failure syndrome: a case–control study

**DOI:** 10.1186/s43044-023-00404-y

**Published:** 2023-09-05

**Authors:** Ahmed Refaat Mohamed Sakr, Gamal Fahim Elsayed Gomaa, Salwa Mahmoud El Wasif, Ahmed Hassan Hosny Eladawy

**Affiliations:** Faculty of Medicine, Mansoura University, Mansoura, Egypt

**Keywords:** Urea-to-creatinine ratio, Acute heart failure, Acute kidney injury, Prognosis

## Abstract

**Background:**

Recent research has shown that the blood urea/creatinine ratio (BUN/Cr) rather than BUN or Cr alone can predict the prognosis of individuals with acute heart failure (AHF). The objective of this study was to estimate the urea-to creatinine serum ratio (BUN/Cr) in patients with acute decompensated heart failure (ADHF) and correlate the results with patient outcome, length of hospitalization, and mortality.

**Results:**

Sixty ADHF patients were included and categorized into four groups; Group I: non-AKI with low BUN/Cr (*n* = 25); Group II: non-AKI with high BUN/Cr (*n* = 5); Group III: AKI with low BUN/Cr (*n* = 14); Group IV: AKI with high BUN/Cr (*n* = 16). Regarding urea and BUN levels, the first reading showed a considerable rise in urea and BUN levels in groups III and IV compared to group 1 and in group IV compared to groups I and III. Similar results were recorded in the second and third readings. Regarding the BUN/Cr ratio, the three readings revealed a significant elevation in group IV compared to groups I and II and in group IV compared to group III. Mortality was significantly higher in group IV compared to group I. Additionally, MACE was significantly more frequent in group IV compared to groups I and III. Multivariable logistic regression analysis revealed that hypertension, creatinine, and BUN were independent predictors of AKI.

**Conclusions:**

BUN/Cr may predict prognosis in AHF patients since AHF with an elevated BUN/Cr is associated with a higher death rate.

## Background

Heart failure (HF) symptoms or indicators can appear gradually or suddenly and are severe enough to demand immediate medical attention or an unplanned hospital stay. Patients with AHF need to be evaluated immediately, followed by the start or intensification of treatments. AHF is a major factor in hospital admissions for patients older than 65 and is linked to high death and readmission rates. Hospital mortality varies between 4 and 10% [1].

The AKI network defines acute kidney injury (AKI) as a rise in absolute serum creatinine of 0.3 mg/dl or a 1.5-fold increase in serum creatinine levels within 48 h [2].

In ADHF patients, AKI is not necessarily associated with mortality. Numerous investigations have demonstrated that elevations in serum creatinine brought on by the alleviation of congestion are not linked to long-term renal impairment or adverse effects [3]. To predict clinical outcomes in ADHF patients, it is crucial to understand the pathways that lead to AKI [3].

In patients with ADHF, renal impairment is a prevalent comorbidity. Higher blood BUN/Cr levels are linked to mortality in patients with concomitant renal impairment and HF [4]. The renal tubules are primarily traversed by creatinine, with little to no reabsorption. Serum creatinine levels are a primary indicator of glomerular filtration rate (GFR) when neurohormonal factors such as arginine, vasopressin, the sympathetic nervous system, and the renin–angiotensin–aldosterone system are all activated [5]. Elevated BUN/Cr, a proxy indicator of severe heart failure, may reveal AKI, which is linked to mortality [6].

When neurohormonal activity is present, creatinine travels through the glomerulus without being reabsorbed, whereas urea is disproportionately reabsorbed, raising the BUN/Cr ratio. Because of this, ADHF patients may experience more negative outcomes when their BUN/Cr is increased than when their creatinine or estimated GFR is elevated [7].

Therefore, the objective of this study was to assess the BUN/Cr in acute decompensated heart failure patients and correlate the results with the outcome, length of hospitalization, and mortality. Additionally, to determine whether clinical markers such as the BUN/Cr ratio or each alone can stratify the mortality risk associated with AKI.

## Methods

This case–control study included 60 patients with acute decompensated heart failure (HFREF Or HFPEF according to EF% calculated by Echocardiography) admitted to the Cardiology Department at Specialized Medical Hospital, Mansoura University, from January to October 2021. Patients were classified into four groups according to AKI and level of Bun/Cr ratio; Group I: non-AKI with low BUN/Cr (*n* = 25); Group II: non-AKI with high BUN/Cr (*n* = 5); Group III: AKI with low BUN/Cr (*n* = 14); Group IV: AKI with high BUN/Cr (*n* = 16). Patients who were less than 18 years, pregnant, required renal replacement therapy, had known obstructive uropathy, or had known renal disease (e.g., polycystic kidney disease, glomerulonephritis) were excluded from the study.

All patients underwent a thorough medical history, chest X-ray, standard supine 12-lead electrocardiography, and echocardiography to detect structural or functional cardiac abnormalities. Blood samples were collected on admission (day one), after 48 h (day two), and before discharge (day three) to carry out laboratory investigations, including complete blood count (CBC), electrolytes (Na+, K+), urea, creatinine, BUN, BUN/Cr, and liver function tests. GFR was calculated by the Cockcroft–Gault formula. Also, coagulation profile (PT, PTT, and INR), cardiac enzymes (CK, CK-MB, and troponin), and arterial blood gases (ABGs) were performed.

Our patients did not take any nephrotoxic medications that may affect renal function and this point was thoroughly investigated during history taking.

We are so cautious for using fluid therapy during management of our patients. CVP cannot reflect volume status in heart failure so we use inotropes and diuretics in 59 patients. Furosemide is the only available diuretic. We used it as infusion for 18 patients in ICU, while the rest of them (41 patients) used it in dose of 10 mg iv/12 h and titrated the dose according to the response, potassium level and patient’s hemodynamics.

### Statistical analysis

SPSS 22.0 for Windows was employed to examine all the data (SPSS Inc., Chicago, IL, USA). The Shapiro–Wilk test was used to assess normality. Frequencies and percentages were used to depict qualitative data. The Chi-square test was employed to determine how qualitative factors differed. Quantitative data were presented as mean ± SD (standard deviation) for parametric data. To compare normally distributed variables between more than two dependent groups, a one-way ANOVA test supplemented with an LSD post hoc test was utilized. ROC analysis was used to evaluate the diagnostic performance of different markers. The area under the curve (AUC) was used to assess the overall performance. The area under the curve of more than 50% represents acceptable performance, and the area of about 100% is the best performance. To determine how two quantitative variables are correlated, the Pearson correlation was used. Statistical comparisons were all two-tailed. The significance level was ≤ 0.05.

## Results

Sixty AHF patients participated in the current research. The majority were males (71.7%). The mean age was 62.8 ± 10.4 years. Based on AKI and BUN/Cr, cases were classified into four groups. Baseline clinical characteristics, co-morbidities, complaints, presentation, HF etiology and treatment, ECG, and ECHO results are outlined in Table 1.

**Table 1:** Baseline characteristics of the patients

Parameter		(*n* = 60)
Age		62.8 ± 10.4
Gender	Male	43 (71.7%)
Co-morbidities		
DM		30 (50.0%)
HTN		37 (61.7%)
COPD		8 (13.3%)
VHD		9 (15.0%)
CAD		14 (23.3%)
Cardiomyopathy		28 (46.7%)
CLD		6 (10.0%)
AF		13 (21.7%)
Smoking		22 (36.7%)
CABG		0 (0.0%)
PCI		4 (6.7%)
Complain		
Orthopnea		47 (78.3%)
Drowsiness		8 (13.3%)
Syncope		10 (16.7%)
Shortness of breath		54 (90.0%)
Palpitation		10 (16.7%)
Chest pain		9 (15.0%)
Presentation		
Acute pulmonary edema		26 (43.3%)
Generalized anasarca		17 (28.3%)
Cardiogenic shock		13 (21.7%)
HF etiology		
ACS		14 (23.3%)
Systemic infection		8 (13.3%)
Severe HTN		4 (6.7%)
Rapid AF		10 (16.7%)
Complete heart block		7 (11.7%)
Valvular heart disease		8 (13.3%)
Anemia		2 (3.3%)
Ventricular arrhythmia		1 (1.7%)
Acute exacerbation of chronic HF		16 (26.7%)
Treatment		
ACEIS		36 (60.0%)
BB		40 (66.7%)
MRA		36 (60.0%)
Diuretics		59 (98.3%)
Inotropics		20 (33.3%)
Vasopressors		13 (21.7%)
ECG		
ST–T wave changes		22 (36.7%)
AF		16 (26.7%)
Complete heart block		7 (11.7%)
Left BBB		19 (31.7%)
LVH		1 (1.7%)
ECHO		
Median EF		39.5 (18–55)
SWMA		25 (41.7%)
Valvular disease		45 (75.0%)
Diastolic dysfunction		10 (16.7%)
Right-sided heart failure		6 (10.0%)

Data are presented as mean ± SD and frequency (%). *DM* Diabetes mellitus, *CAD* Coronary artery disease, *HTN* Hypertension, *COPD* Chronic obstructive pulmonary disease, *VHD* Valvular heart disease, *CLD* Chronic liver disease, *AF* Atrial fibrillation

Table 2 shows significantly higher NYHA (4) in the AKI group versus the non-AKI group. SBP and DBP were significantly elevated in the non-AKI group compared to the AKI group. AKI patients had a significantly longer hospital stay than those without AKI. The non-AKI patients were more frequently treated by ACEIS, BB, and MRA, while the AKI group was more frequently treated by vasopressors. The AKI group had significantly higher creatinine, urea, BUN, and BUN/Cr in the first, second, and third readings than the non-AKI patients. GFR was significantly decreased in the first, second, and third readings in the AKI group versus the non-AKI group. K levels in the AKI group were substantially higher than in the non-AKI group (Table 2).

**Table 2:** Comparison of NYHA, EF, blood pressure, treatment, and laboratory findings between patients with and without AKI

Parameter	Non-AKI (*n *= 30)	AKI (*n* = 30)	*P* value
NYHA class D1			
1	0 (0.0%)	0 (0.0%)	0.046
2	0 (0.0%)	1 (3.3%)	
3	12 (40.0%)	4 (13.3%)	
4	18 (60.0%)	25 (83.3%)	
NYHA class D2			
1	1 (3.3%)	2 (6.7%)	0.014
2	15 (50.0%)	6 (20.0%)	
3	13 (43.3%)	13 (43.3%)	
4	1 (3.3%)	9 (30.0%)	
NYHA class D3			
1	6 (20.0%)	5 (16.7%)	0.017
2	21 (70.0%)	12 (40.0%)	
3	3 (10.0%)	7 (23.3%)	
4	0 (0.0%)	6 (20.0%)	
SBP*	110.0 (60–210)	100.0 (60–200)	0.026
DBP*	70.0 (30–110)	60.0 (40–100)	0.032
EF*	40.0 (18–55)	38.5 (19–55)	0.486
Length of stay in hospital*	4.5 (3–18)	8.5 (3–29)	< 0.001
Treatment			
ACEIS	27 (90.0%)	9 (30.0%)	< 0.001
BB	24 (80.0%)	16 (53.3%)	0.028
MRA	26 (86.7%)	10 (33.3%)	< 0.001
Diuretics	30 (100.0%)	29 (96.7%)	1.00
Inotropics	8 (26.7%)	12 (40.0%)	0.273
Vasopressors	1 (3.3%)	12 (40.0%)	0.001
HCT*	35.5 ± 5.7	34.1 ± 6.3	0.373
Creatinine (first reading)	1.3 (0.7–2.9)	2.5 (0.7–5.9)	< 0.001
Creatinine (second reading)	1.3 (1.0–3.0)	3.0 (2.0–6.0)	< 0.001
Creatinine (third reading)	1.1 (0.8–1.9)	2.8 (0.9–5.8)	< 0.001
Urea (first reading)	43.5 (22–88)	98.0 (31–210)	< 0.001
Urea (second reading)	46.5 (28–157)	136.5 (20–280)	< 0.001
Urea (third reading)	38.0 (21–95)	100.0 (30–290)	< 0.001
BUN (first reading)	21.0 (10.2–65.4)	45.7 (14.4–98.0)	< 0.001
BUN (second reading)	21.5 (13–73)	63.5 (19–130)	< 0.001
BUN (third reading)	17.4 (10.5–44.3)	46.0 (14–135.5)	< 0.001
BUN/Cr (first reading)	15.4 (10.5–41.4)	21.1 (7.7–37.0)	0.013
BUN/Cr (second reading)	15.0 (10–27)	17.0 (8.0–37)	0.048
BUN/Cr (third reading)	14.9 (11.6–26.2)	19.5 (4.7–32.3)	0.046
GFR (first reading)	76.8 (28.1–144)	34.4 (14.1–167.0)	< 0.001
GFR (second reading)	66.0 (24–123)	30.0 (12–73)	< 0.001
GFR (third reading)	83.0 (36–115)	30.5 (10.1–110.2)	< 0.001
Albumin	3.9 (3.0–4.8)	3.8 (1.2–4.5)	0.047
INR	1.0 (1.0–2.0)	1.0 (1.0–3.0)	0.113
Na	124.0 (120–128)	123.5 (120–128)	0.759
K	4.0 (2.9–6.0)	5.0 (3.0–6.0)	0.016

Data are presented as mean ± SD, median (Min–Max), and frequency (%). *P* < 0.05 is considered significant. Glomerular filtration rate: GFR, BUN: Blood urea nitrogen, Cr: Creatinine, Na: Sodium, K: Potassium, INR: International normalized ratio

*Statistically significant, independent predictor

**Fig. 1:**
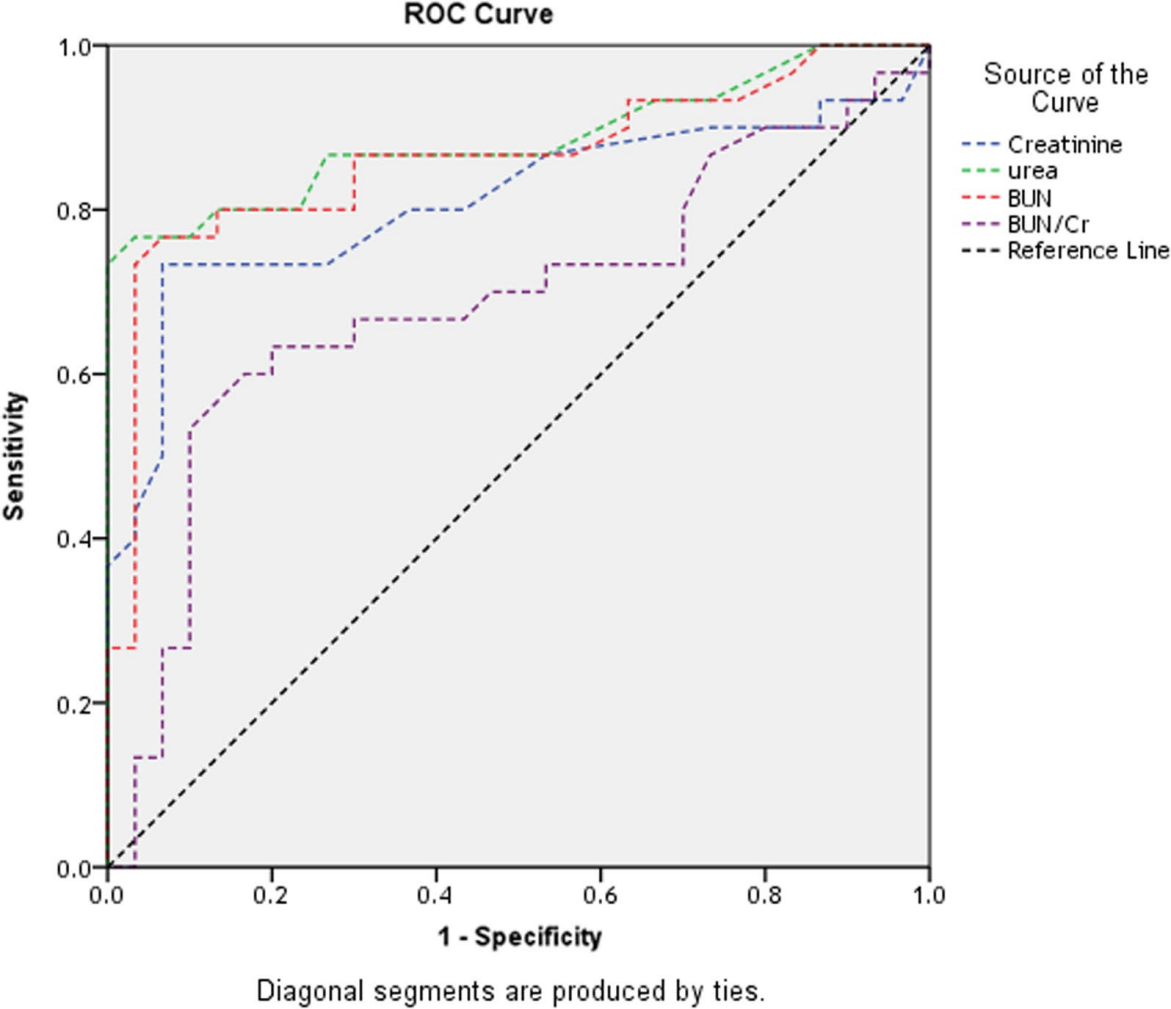
ROC curve analysis

ROC analysis was used to determine the optimal cutoff levels for predicting AKI. The creatinine best cutoff value was 1.85, with an AUC of 0.811 (*P* < 0.001). The urea best cutoff value was 64.0, with an AUC of 0.866 (*P* < 0.001). The BUN best cutoff value was 31.5, with an AUC of 0.862 (*P* < 0.001). The BUN/Cr best cutoff value was 17.4, with an AUC of 0.687 (*P* = 0.013) (Fig. 1).

Table 3 shows a significant increase in NYHA class 4 on day 1 in group IV compared to group I and on days 2 and 3 in group IV compared to groups I and III. There was a significant decrease in SBP and DBP in group IV compared to groups I and III. The length of hospital stay was significantly higher in groups III and IV compared to group 1. Additionally, drug use significantly differed among the four groups (Table 3).

**Table 3:** Comparison of NYHA, EF, blood pressure, and treatment among the studied groups regarding the presence and absence of AKI with low or high BUN/Cr

Parameter	Group 1 (*n* = 25)	Group 2 (*n* = 5)	Group 3 (*n* = 14)	Group 4 (*n* = 16)	*P *value
NYHA class D1					
1	0 (0.0%)	0 (0.0%)	0 (0.0%)	0 (0.0%)	*P* = 0.135 *P*^3^ = 0.034
2	0 (0.0%)	0 (0.0%)	1(7.1%)	0 (0.0%)	
3	11 (44.0%)	1 (20.0%)	2 (14.3%)	2 (12.5%)	
4	14 (56.0%)	4 (80.0%)	11 (78.6%)	14 (87.5%)	
NYHA class D2					
1	1 (4.0%)	0 (0.0%)	2 (14.3%)	0 (0.0%)	*P* = 0.004 *P*^3^ = 0.001 *P*^6^ = 0.017
2	13 (52.0%)	2 (40.0%)	5 (35.7%)	1 (6.2%)	
3	10 (40.0%)	3 (60.0%)	6 (42.9%)	7 (43.8%)	
4	1 (4.0%)	0 (0.0%)	1 (7.1%)	8 (50.0%)	
NYHA class D3					
1	6 (24.0%)	0 (0.0%)	4 (28.7%)	1 (6.2%)	*P* = 0.005 *P*^3^ ≤ 0.001 *P*^6^ = 0.025
2	17 (68.0%)	4 (80.0%)	8 (57.1%)	4 (25.0%)	
3	2 (8.0%)	1 (20.0%)	1 (7.1%)	6 (37.5%)	
4	0 (0.0%)	0 (0.0%)	1 (7.1%)	5 (31.3%)	
SBP*	120.0 (60–210)	100.0 (80–140)	115.0 (70–200)	80.0 (60–160)	*P* = 0.332 *P*^3^ = 0.001 *P*^6^ = 0.010
DBP*	70.0 (30–110)	70.0 (50–90)	70.0 (40–100)	55.0 (40–100)	*P* = 0.362 *P*^3^ = 0.001 *P*^6^ = 0.004
EF*	40.0 (18–55)	40.0 (27–50)	39.5 (20–55)	38.0 (19–55)	0.988
Length of hospital stay *	4.0 (3–18)	8.0 (7–18)	8.0 (3–23)	10.5 (7–29)	*P* ≤ 0.001 *P*^1^ = 0.002 *P*^2^ = 0.010 *P*^3^ ≤ 0.001
ACEIS	23 (92.0%)	4 (80.0%)	5 (35.7%)	4 (25.0%)	*P* ≤ 0.001 *P*^2^ ≤ 0.001 *P*^3^ ≤ 0.001 *P*^5^ = 0.027
BB	22 (88.0%)	2 (40.0%)	9 (64.3%)	7 (43.8%)	*P* = 0.015 *P*^1^ = 0.014 *P*^3^ = 0.002
MRA	23 (92.0%)	3 (60.0%)	6 (42.9%)	4 (25.0%)	*P* ≤ 0.001 *P*^2^ ≤ 0.001 *P*^3^ ≤ 0.001
Diuretics	25 (100.0%)	5 (100.0%)	13 (92.3%)	16 (100.0%)	0.342
Inotropics	3 (12.0%)	5 (100.0%)	3 (21.4%)	9 (56.2%)	*P* ≤ 0.001 *P*^1^ ≤ 0.001 *P*^3^ = 0.002 *P*^4^ = 0.002
Vasopressors	0 (0.0%)	1 (20.0%)	3 (21.4%)	9 (56.2%)	*P* ≤ 0.001 *P*^1^ ≤ 0.001 *P*^2^ = 0.015 *P*^3^ ≤ 0.001

Data are presented as mean ± SD, median (Min–Max), and frequency (%). *P* < 0.05 is considered significant. ^DBP: Diastolic blood pressure, SBP: Systolic blood pressure, EF: Ejection fraction, ACEIS: Angiotensin-converting enzymes inhibitors, BB: Beta blockers, MRA: Mineralocorticoid receptor antagonists. P between 4 groups. P1 between group 1 and group 2, P2 between group 1 and group 3. P3 between group 1 and group 4. P4 between group 2 and group 3. P5 between group 2 and group 4. P6 between group 3 and group 4^

*Statistically significant, independent predictor

Table 4 demonstrates a significant increase in creatinine levels at the first and third readings in groups III and IV compared to group I, and in group IV compared to group I. Similar results were recorded in the second reading.

**Table 4:** Comparison of laboratory parameters among patient group regarding presence and absence of AKI with low or high BUN/Cr

Parameter	Group 1 (*n* = 25)	Group 2 (*n* = 5)	Group 3 (*n* = 14)	Group 4 (*n* = 16)	*P* value
Hb*	12.5 ± 2.22	12.1 ± 2.23	11.9 ± 3.38	11.8 ± 1.72	0.816
HCT*	35.7 ± 5.69	34.6 ± 6.65	34.14 ± 8.37	34.13 ± 4.03	0.821
Creatinine (1st reading)	1.3 (0.8–2.9)	1.2 (0.7–2.3)	2.8 (0.7–5.9)	2.1 (1.3–4.3)	*P* = 0.058 *P*^2^ = 0.026 *P*^3^ ≤ 0.001 *P*^5^ = 0.025
Creatinine (2nd reading)	1.0 (1.0–2.0)	2.0 (1.0–3.0)	3.5 (2.0–6.0)	3.0 (2.0–6.0)	*P* ≤ 0.001 *P*^2^ ≤ 0.001 *P*^3^ ≤ 0.001 *P*^4^ = 0.034 *P*^5^ = 0.015
Creatinine (3rd reading)	1.1 (0.9–1.8)	1.6 (0.8–1.9)	2.6 (0.9–5.1)	3.05 (1.2–5.8)	*P* = 0.004 *P*^2^ = 0.001 *P*^3^ ≤ 0.001 *P*^5^ = 0.008
Urea (1st reading)	42.0 (22–70)	48.0 (28.1–88)	90.0 (31–170)	115.0 (68–210)	*P* = 0.019 *P*^2^ = 0.006 *P*^3^ ≤ 0.001 *P*^5^ ≤ 0.001 *P*^6^ = 0.017
Urea (2nd reading)	44.0 (28–82)	85.0 (38.0–157.0)	93.5 (41–195)	150.0 (20–280)	*P* ≤ 0.001 *P*^1^ = 0.013 *P*^2^ ≤ 0.001 *P*^3^ ≤ 0.001 *P*^5^ = 0.050 *P*^6^ = 0.007
Urea (3rd reading)	35.0 (21–67)	70.0 (37–95)	74.0 (30–140)	155.0 (52–290)	*P* = 0.003 *P*^1^ = 0.004 *P*^2^ = 0.005 *P*^3^ ≤ 0.001 *P*^5^ = 0.004 *P*^6^ = 0.001
BUN (1st reading)	20.0 (10.2–32.7)	37.3 (14–65.4)	42.0 (14.4–79.0)	53.5 (32.0–98.0)	*P* = 0.013 *P*^2^ = 0.006 *P*^3^ ≤ 0.001 *P*^5^ = 0.019 *P*^6^ = 0.017
BUN (2nd reading)	20.0 (13–32)	40.0 (18–73)	43.5 (19–91)	70.0 (36–130)	*P* ≤ 0.001 *P*^1^ = 0.013 *P*^2^ ≤ 0.001 *P*^3^ ≤ 0.001 *P*^5^ = 0.015 *P*^6^ = 0.002
BUN (3rd reading)	16.3 (10.5–31.3)	32.0 (17.2–44.3)	34.5 (14–65)	72 (22.5–135.5)	*P* = 0.002 *P*^1^ = 0.003 *P*^2^ = 0.005 *P*^3^ ≤ 0.001 *P*^5^ = 0.004 *P*^6^ = 0.001
BUN/Cr (1st reading)	15.0 (10.5–19.6)	24.0 (18.6–41.4)	13.6 (7.7–26.0)	23.7 (15.5–37.0)	*P* = 0.005 *P*^1^ ≤ 0.001 *P*^3^ ≤ 0.001 *P*^4^ = 0.007 *P*^6^ ≤ 0.001
BUN/Cr (2nd reading)	15.0 (10.0–20.0)	21.0 (17.0–27.0)	12.5 (8.0–18.0)	23.5 (15.0–37.0)	*P* ≤ 0.001 *P*^1^ ≤ 0.001 *P*^3^ ≤ 0.001 *P*^4^ ≤ 0.001 *P*^6^ ≤ 0.001
BUN/Cr (3rd reading)	14.3 (11.6–24.1)	21.5 (18.4–26.2)	13.9 (4.7–17.1)	22.1 (18.5–32.3)	*P* = 0.002 *P*^1^ ≤ 0.001 *P*^3^ ≤ 0.001 *P*^4^ ≤ 0.001 *P*^6^ ≤ 0.001
GFR (1st reading)	76.7 (29.3–127.0)	104.0 (28.1–144)	29.0 (15–167)	40.5 (14.1–102)	*P* = 0.041 *P*^2^ = 0.017 *P*^3^ ≤ 0.001 *P*^5^ = 0.025
GFR (2nd reading)	66.0 (37–115)	52.0 (24–123)	25.0 (14–68)	31.0 (12–73)	*P* = 0.001 *P*^2^ ≤ 0.001 *P*^3^ ≤ 0.001 *P*^5^ = 0.032
GFR (3rd reading)	82.0 (47.5–115)	85.0 (36.0–114)	32.5 (17.1–106)	27.0 (10.1–110.2)	*P* = 0.005 *P*^2^ = 0.001 *P*^3^ ≤ 0.001 *P*^4^ = 0.044 *P*^5^ = 0.015
Albumin	3.9 (3–4.8)	3.8 (3.2–4.5)	3.8 (1.2–4.5)	3.5 (2.9–4.1)	*P* = 0.619 *P*^3^ = 0.017
INR	1.0 (1–2)	1.0 (1–2)	1.0 (1–2)	1.0 (1–3)	0.278
Na	124.0 (120–128)	124.0 (120–128)	123.5 (120–126)	123.5 (120–128)	0.940
K	4.0 (2.9–6.0)	4.0 (4–6)	5.0 (4–6)	4.5 (3–6)	*P* = 0.009 *P*^2^ = 0.008

Data are presented as mean ± SD, median (Min–Max), and frequency (%). *P* < 0.05 is considered significant. GFR: Glomerular filtration rate, K: Potassium, Cr: Creatinine, Na: Sodium, BUN: Blood urea nitrogen, INR: International normalized ratio

*Statistically significant, independent predictor

Regarding urea and BUN levels, the first reading revealed a significant increase in groups III and IV compared to group I, and in group IV compared to groups I and III. A similar pattern was reported in the second and third urea and BUN readings.

Regarding the BUN/Cr ratio, the three readings revealed a significant elevation in group IV compared to groups I and II, and in group IV compared to group III. Additionally, the GFR significantly declined in groups III and IV compared to group I.

Albumin level significantly declined in group IV compared to group I, while K level was significantly higher in group III compared to group 1 **(**Table 4).

Table 5 shows that death was significantly more frequent in group IV than in group I, while MACE was significantly more frequent in group IV compared to groups I and III (Table 5).

**Table 5:** Comparison of outcomes among the four groups regarding the presence and absence of AKI with low or high BUN/Cr

Parameter	Group 1 (*n* = 25)	Group 2 (*n* = 5)	Group 3 (*n* = 14)	Group 4 (*n* = 16)	*P* value
Death	1 (4.0%)	1 (20.0%)	3 (21.4%)	9 (56.2%)	*P* = 0.001 *P*^3^ ≤ 0.001
HD	0 (0.0%)	0 (0.0%)	1 (7.1%)	2 (12.5%)	0.307
MACE	1 (4.0%)	2 (40.0%)	1 (7.1%)	7 (43.8%)	*P* = 0.004 *P*^1^ = 0.014 *P*^3^ = 0.001 *P*^6^ = 0.026

HD: Hemodialysis, MACE: Major adverse cardiac event. One-way ANOVA*, Chi-square test. P between 4 groups. P1 between group 1 and group 2, P2 between group 1 and group 3. P3 between group 1 and group 4. P4 between group 2 and group 3. P5 between group 2 and group 4. P6 between group 3 and group 4

The AKI risk was predicted using logistic regression analysis. The covariates included HTN, SBP, DBP, creatinine, urea, BUN, BUN/Cr, GFR, albumin, and K. Only HTN, creatinine, and BUN were considered independent AKI predictors after the inclusion of significant factors at the univariate level in a multivariable analysis **(**Table 6).

**Table 6:** Regression analysis for prediction of AKI

	Univariable				Multivariable			
	*P*	OR	95% CI		*P*	OR	95% CI	
History of HTN	0.019	3.755	1.239	11.385	0.048	7.135	1.017	50.059
SBP	0.037	0.985	0.970	0.999	0.980	0.999	0.944	1.058
DBP	0.038	0.972	0.947	0.999	0.861	0.990	0.890	1.103
Creatinine	0.001	5.758	2.040	16.248	0.046	2.594	1.084	30.269
Urea	< 0.001	1.064	1.032	1.097	0.308	1.095	0.919	1.305
BUN	< 0.001	1.105	1.050	1.163	0.039	1.044	1.021	1.385
BUN/Cr	0.030	1.103	1.009	1.206	0.996	1.001	0.769	1.302
GFR	0.003	0.972	0.954	0.990	0.186	0.990	0.968	1.063
Albumin	0.053	0.334	0.110	1.015	–	–	–	–
K	0.040	2.004	1.034	3.883	0.128	2.462	0.771	7.865

OR: odds ratio; CI, confidence interval; logistic regression analysis was used. *P* < 0.05 indicates statistical significance. K: Potassium, HTN: Hypertension^, DBP: Diastolic blood pressure, SBP: Systolic blood pressure^, Na: Sodium, BUN: Urea nitrogen, BUN/Cr: Urea/creatinine ratio

## Discussion

The current study aimed to investigate if clinical markers such as the BUN/Cr ratio, BUN, or creatinine can stratify the AKI mortality risk in ADHF patients and whether these markers are associated with the outcome, length of hospital stay, and death.

This study observed a significant increase in NYHA class 4 on day 1 in group IV compared to group I and on days 2 and 3 in group IV compared to groups I and III. There was a substantial decrease in SBP and DBP in group IV compared to groups I and III. In line, Takaya et al. [2] reported that compared to the other groups, blood pressure (systolic and diastolic) is lower in group 4, and it is usually treated with IV dopamine and dobutamine therapy.

Our study demonstrated a significant increase in creatinine at the first and third readings in groups III and IV compared to group I. Similar findings were observed in the second reading, with a significant elevation in group III compared to group I.

First readings of urea and BUN showed a significant rise in groups III and IV compared to group I, and in group IV compared to groups I and III. Comparable results were reported in the second and third readings.

The BUN/Cr ratio readings demonstrated a significant rise in group IV compared to groups I, II, and III. There was a substantial decline in GFR in groups III and IV compared to group I in the three GFR readings. These results are compatible with Takaya et al. [2], who reported that AKI patients with high BUN/Cr have lower GFR but higher BUN, creatinine, and BUN/Cr levels than the other groups.

Higher creatinine levels may be due to either permanent renal damage or congestion relief. On the other hand, urea excretion is decreased by renal vasoconstriction and decreased GFR caused by neurohormonal components, such as the sympathetic nervous system and renin–angiotensin–aldosterone system [8, 9].

Additionally, flow- and concentration-dependent urea absorption is increased by neurohormonal activity [10]. Low cardiac output causes arterial underfilling that induces arginine vasopressin production, encouraging urea reabsorption [10]. While urea is disproportionately reabsorbed during neurohormonal activation, causing an increased BUN/Cr ratio, the glomerulus freely filters creatinine, which is not reabsorbed. [11]. Therefore, a higher BUN/Cr more accurately represents neurohormonal activity than a higher creatinine or a lower estimated GFR [2].

In regression analysis for predicting AKI, only HTN, creatinine, and BUN were independent predictors of AKI. Takaya et al. [2] observed that BUN, creatinine, and intravenous dobutamine are independent risk factors for AKI. Additionally, Tung et al. [12] stated that age, GFR, WBCS, Hb, BUN, creatinine, B-type natriuretic peptide, Cystatin C, and neutrophil gelatinase-associated lipocalin are associated with AKI in the univariate analysis. However, no variable remained significant after multivariate analysis.

The current study has some limitations: the first is the observational nature of the study. Second, the non-neurohormonal factors affecting the BUN/Cr ratio include a high-protein diet, cachexia, and muscular atrophy; however, these elements were not examined in this investigation. Third, the time interval for serum creatinine tests was not just 48 h since the timing of laboratory measurements was allowed at the treating physicians' discretion, thereby underestimating the incidence of AKI. Finally, the AKI network criteria use urine output and serum creatinine to define AKI; however, we only used serum creatinine. Therefore, these results need to be supported by data from sizable, well-planned trials to advance our understanding of the BUN/Cr in AHF patients.

## Conclusions

In ADHF patients, higher mortality risk is associated with AKI and an increased BUN/Cr on admission, but not with BUN or creatinine alone. Our results imply that the BUN/Cr on admission determines AKI prognosis and is helpful for risk stratifying. AKI risk assessment, which can be completed on admission, may help decide whether to continue decongestion therapy for ADHF patients with low BUN/Cr despite elevated creatinine levels as opposed to considering additional ADHF treatment options in AKI patients with a high BUN/Cr. Additional research is required to verify these results and explore therapeutic approaches to enhance clinical outcomes in ADHF patients.

## Data Availability

Data and material are available on a reasonable request from the author.

## Abbreviations

AHF: Acute heart failure
BUN/Cr: Urea-to-creatinine serum ratio
AKI: Acute kidney infarction
HF: Heart failure
GFR: GFR
ROC: Receiver operating characteristic curve
ADHF: Acute decompensated heart failure
DM: Diabetes mellitus
HTN: Hypertension
COPD: Chronic obstructive pulmonary disease
VHD: Valvular heart disease
CAD: Coronary artery disease
CLD: Chronic liver disease
AF: Atrial fibrillation
NYHA: New York Heart Association
SBP: Systolic blood pressure
DBP: Diastolic blood pressure
EF: Ejection fraction
INR: International normalized ratio
Na: Sodium
K: Potassium
ACEIS: Angiotensin-converting enzymes inhibitors
BB: Beta blockers
MRA: Mineralocorticoid receptor antagonists
HD: Hemodialysis
MACE: Major adverse cardiac event
